# Assessment of quantitative information for radiation therapy at a first-generation clinical photon-counting computed tomography scanner

**DOI:** 10.3389/fonc.2022.970299

**Published:** 2022-09-14

**Authors:** Guyue Hu, Katharina Niepel, Franka Risch, Christopher Kurz, Matthias Würl, Thomas Kröncke, Florian Schwarz, Katia Parodi, Guillaume Landry

**Affiliations:** ^1^ Department of Medical Physics, Faculty of Physics, Ludwig-Maximilians-Universität München (LMU), Garching bei München, Germany; ^2^ Department of Diagnostic and Interventional Radiology, Universitätsklinikum Augsburg, Augsburg, Germany; ^3^ Department of Radiation Oncology, LMU Klinikum, Munich, Germany; ^4^ Medical Faculty, Ludwig-Maximilians-Universität München, Munich, Germany; ^5^ German Cancer Consortium (DKTK), Partner Site Munich, Munich, Germany

**Keywords:** photon-counting CT, relative stopping power (RSP), proton therapy, virtual monoenergetic images (VMIs), relative electron density, effective atomic number

## Abstract

As one of the latest developments in X-ray computed tomography (CT), photon-counting technology allows spectral detection, demonstrating considerable advantages as compared to conventional CT. In this study, we investigated the use of a first-generation clinical photon-counting computed tomography (PCCT) scanner and estimated proton relative (to water) stopping power (RSP) of tissue-equivalent materials from virtual monoenergetic reconstructions provided by the scanner. A set of calibration and evaluation tissue-equivalent inserts were scanned at 120 kVp. Maps of relative electron density (RED) and effective atomic number (EAN) were estimated from the reconstructed virtual monoenergetic images (VMI) using an approach previously applied to a spectral CT scanner with dual-layer detector technology, which allows direct calculation of RSP using the Bethe-Bloch formula. The accuracy of RED, EAN, and RSP was evaluated by root-mean-square errors (RMSE) averaged over the phantom inserts. The reference RSP values were obtained experimentally using a water column in an ion beam. For RED and EAN, the reference values were calculated based on the mass density and the chemical composition of the inserts. Different combinations of low- and high-energy VMIs were investigated in this study, ranging from 40 to 190 keV. The overall lowest error was achieved using VMIs at 60 and 180 keV, with an RSP accuracy of 1.27% and 0.71% for the calibration and the evaluation phantom, respectively.

## 1 Introduction

Proton therapy allows for a highly conformal dose to the tumor region, sparing the surrounding healthy tissue (1). On the other hand, it requires accurate prediction of proton range, which is typically determined by the proton relative (to water) stopping power (RSP). In current clinical practice, RSP is estimated using single-energy X-ray computed tomography (CT) by conversion from CT numbers measured in Hounsfield units (HU). The relations between CT numbers and RSP are not unique, causing uncertainties for the semi-empirical HU-RSP conversion (2). Proton range uncertainties of 3.5% have been reported (3). In contrast, dual-energy CT (DECT), using two different energy spectra, makes it possible to directly estimate the relative electron density (RED) and effective atomic number (EAN) of materials from CT images acquired at different energies, and subsequently calculate the proton RSP. This approach thus potentially reduces RSP estimation errors and range prediction uncertainties (4–8). DECT has recently seen clinical implementation, and has led to the reduction of range uncertainty safety margins to 2% in at least two institutions (9, 10). The technique has also been compared favorably to direct measurement approaches under development such as proton or ion CT (11, 12).

Photon-counting detector technology, in which individual photons are counted in selected energy bins, is one of the latest developments in X-ray CT (13–15). Photon-counting CT (PCCT) datasets allow multi-energy processing beyond what is feasible with DECT. Early work using a prototype PCCT scanner reported that performance was on par with DECT (16). Recently, first-generation clinical PCCT scanners have been clinically introduced. However, these scanners do not yet come with software allowing the extraction of quantitative information used for radiation therapy treatment planning, such as RED, EAN, and RSP, and their performance has not been investigated yet. In this work, we aim at evaluating a first-generation clinical PCCT scanner with an approach relying on the virtual monoenergetic images (VMIs) which can be reconstructed using the standard scanner software and are available to all users. Using these images, RED, EAN, and RSP were derived for two phantoms containing tissue-equivalent inserts and compared to reference values.

## 2 Materials and methods

### 2.1 Phantom scans

The measurements were performed on a new clinical PCCT scanner (Siemens NAEOTOM Alpha, *Siemens Healthineers, Forchheim, Germany*) at a dose level of CTDI_vol_=40 mGy (measured in a head dosimetry phantom with a diameter of 16 cm) with dose modulation turned off, a collimation width of 144 × 0.4mm, and a pitch of 0.55. A calibration phantom (Ø 150 mm) containing twelve Gammex 467 inserts (*Gammex, Inc., Middleton, WI, USA*) and an evaluation phantom (Ø 130 mm) containing six CIRS 062M inserts (*CIRS, Inc., Norfolk, VA, USA*), as listed in **Table 1**, were scanned at a tube voltage of 120 kVp using a four energy bins mode which are combined to yield two basis images. The four energy thresholds are manufacturer-specified. Subpixels are read out in 2×2 groups for this mode (17). The clinical scanner software does not allow direct access to the reconstructed basis images used in Taasti et al. (16) for RSP estimation. We thus made use of VMIs which are easily accessible. VMIs were reconstructed with the *Qr36f* kernel at energies ranging from 40 keV to 190 keV in 10 keV increments, resulting in 512×512-pixel images with 0.38 × 0.38mm^2^ pixel spacing and 1 mm slice thickness.

**Table 1:** Mass percent elemental composition and mass density of insert materials.

Element	*ρ*	H	C	N	O	Mg	Si	P	Cl	Ca
Z	[g cm^-3^]	1	6	7	8	12	14	15	17	20
A		1.008	12.01	14.01	16.00	24.31	28.09	30.97	25.45	40.08
I		19.2	81	82	106	176	195	195	180	216
**Gammex**										
BR-12 Breast	0.98	8.59	70.11	2.33	17.90	0	0	0	0.13	0.95
AP6 Adipose	0.94	9.06	72.30	2.25	16.27	0	0	0	0.13	0
LN-450 Lung	0.45	8.47	59.79	1.97	18.11	11.21	0.58	0	0.10	0
LN-300 Lung	0.30	8.46	59.38	1.96	18.14	11.19	0.78	0	0.10	0
LV1 Liver	1.10	8.06	67.01	2.47	20.01	0	0	0	0.14	2.31
BRN-SR2 Brain	1.05	10.83	72.54	1.69	14.86	0	0	0	0.08	0
Muscle	1.05	8.10	67.17	2.42	19.85	0	0	0	0.14	2.32
CT Solid Water	1.02	8.02	67.23	2.41	19.91	0	0	0	0.14	2.31
CB2-50% CaCO3	1.56	4.77	41.63	1.52	32.00	0	0	0	0.08	20.02
CB2-30% CaCO3	1.34	6.68	53.48	2.12	25.61	0	0	0	0.11	12.01
B200 Bone Mineral	1.15	6.65	55.52	1.98	23.64	0	0	3.24	0.11	8.87
IB Inner Bone	1.14	6.67	55.64	1.96	23.52	0	0	3.23	0.11	8.86
**CIRS**										
Liver	1.07	9.00	69.40	2.10	17.10	0	0	0	0.10	2.20
Cortical Bone	1.91	3.30	25.37	0.91	35.28	3.36	0	8.82	0.03	22.91
Muscle	1.06	9.10	69.70	2.10	16.80	0	0	0	0.10	2.20
Adipose	0.96	10.00	71.30	1.80	16.40	0	0	0	0.20	0.30
Trabecular Bone 200	1.16	7.00	56.30	2.00	22.70	0	0	3.30	0.20	8.50
Breast 50/50	0.99	9.60	70.30	1.90	17.00	0	0	0	0.20	0.90

### 2.2 Conversion of PCCT data

Following the approach adopted by (18), RED (*ρ*_e_ ), EAN (*Z*_eff_ ), and RSP were converted from PCCT data using the VMIs and the formalism proposed by Saito and Sagara (19) implemented in in-house software. RED, represented by *ρ*_e_ , was derived using results of calibrations with mean CT numbers of Gammex inserts based on each pair of high- and low-energy VMIs, denoted by HU_H_ and HU_L_ (20):


(1) $$\rho_{\text{e}}=a\frac{(1+\alpha )HU_{H}-\alpha HU_{L}}{1000}+b,$$

where *a* , *b* , and *α* were obtained by least-squares fitting of *ρ*_e_ of the Gammex inserts to a weighted subtraction of CT numbers from high- and low-energy VMIs.

In order to estimate EAN, denoted by *Z*_eff_ , the ratio of EAN of Gammex insert materials to that of water was fitted to the reduced CT number $u_{\text{L}}=\frac{HU_{L}}{1000}+1$ and RED (19):


(2) $${\left(\frac{Z_{eff}}{Z_{eff,w}}\right)}^{m}-1=\gamma_{\text{L}}\left(\frac{u_{L}}{\rho_{e}}-1\right)+\gamma_{0},$$

where *γ*_L_ and *γ*_0_ were obtained by least-squares fitting. EAN was evaluated by Mayneord’s equation with an exponent *m*=3.3 (21, 22):


(3) $$Z_{\text{eff}}={\left(\frac{\sum_{i}\frac{\omega_{i}Z_{i}}{A_{i}}Z_{i}^{m}}{\sum_{i}\frac{\omega_{i}Z_{i}}{A_{i}}}\right)}^{1/m},$$

where *ω*_*i*_ represents the mass weight of the composing element with an atomic number of *Z*_*i*_ and an atomic mass of *A*_*i*_ . The elemental composition of the insert materials are listed in **Table 1**.

Mean excitation energy *I* was calibrated against EAN for soft-tissue and bone-tissue inserts, respectively, with a separation point at EAN=8.8 (21):


(4) $$\ln\;\frac{I}{I_{w}}=C_{1}^{\text{soft}/\text{bone}}\left[{\left(\frac{Z_{eff}}{Z_{eff,w}}\right)}^{m}-1\right]-C_{0}^{\text{soft}/\text{bone}},$$

where *I*_w_=78 eV is the mean excitation energy of water recommended by ICRU report 90 (23, 24); the mean excitation energy of the studied inserts is defined as (21):


(5) $$\ln\;I=\frac{\sum_{i}\frac{\omega_{i}Z_{i}}{A_{i}}ln\;I_{i}}{\sum_{i}\frac{\omega_{i}Z_{i}}{A_{i}}}.$$

Two sets of $C_{1}^{\text{soft}/\text{bone}}$ and $C_{0}^{\text{soft}/\text{bone}}$ were obtained with least-squares fitting for soft-tissue and bone-tissue inserts, respectively.

In this study, we exploited various pairs of monoenergies from the reconstructed VMIs ranging from 40 keV to 190 keV in 10 keV increments. For VMIs at each low energy *E*_L_, a sequence of high energies ranging from *E*_L_+10 to 190keV was selected, which resulted in 105 different energy pairs. For each high- and low-energy pair, the measured CT numbers were converted to RED, EAN, and mean excitation energy using the obtained fit parameters. RSP of each voxel was calculated from the RED and mean excitation energy by the Bethe-Bloch formula (21):


(6) $$\text{RSP}=\rho_{\text{e}}\frac{ln\;\frac{2m_{e}c^{2}\beta^{2}}{I(1-\beta^{2})}-\beta^{2}}{ln\;\frac{2m_{e}c^{2}\beta^{2}}{I_{w}(1-\beta^{2})}-\beta^{2}},$$

where *m*_e_ is electron mass, c the speed of light, and *β* the velocity relative to the speed of light.

### 2.3 Evaluation of RED, EAN, and RSP accuracy

Mean values of the estimated RED, EAN, and RSP were calculated in a region of interest (ROI) for each insert. For each insert, which has a diameter of 29mm (Gammex) or 30mm (CIRS), a cylindrical ROI covering the central volume of the insert was used, with a margin of 5 pixels (i.e., 1.9mm) excluding voxels at the borders.

In order to evaluate the accuracy of RED, EAN, and RSP, the mean estimated values for the calibration and evaluation inserts were compared to their reference values. The reference RED values were calculated from the mass density *ρ* of the inserts provided by the manufacturers:


(7) $$\rho_{\text{e},\text{ref}}=\rho \cdot \frac{\sum_{i}\omega_{i}Z_{i}/A_{i}}{\omega_{H}Z_{H}/A_{H}+\omega_{O}Z_{O}/A_{O}},$$

where the denominator is the weighted sum of *Z*/*A* for composing elements of water. Equation 3 was used to obtain reference values of EAN. The weighting factors *ω*_*i*_ , atomic numbers *Z*_*i*_ , atomic masses *A*_*i*_ , and mean excitation energies *I*_*i*_ in equations 3, 5, and 7 used in this work are tabulated in **Table 1**.

For RSP, the reference values were experimentally assessed through PEAKFINDER (*PTW, Freiburg, Germany*) water column measurements in a carbon ion beam for all the inserts in this study except the Gammex insert *Muscle*, as reported in Hudobivnik et al. (25). For RSP accuracy calculations the *Muscle* insert was thus omitted.

For each high- and low-energy pair, root-mean-square errors (RMSE) of the estimated RED, EAN, and RSP were evaluated for the calibration and the evaluation phantom, respectively, using the following equations:


(8) $$\text{residuals}=100\%\cdot \frac{value_{est}-value_{ref}}{value_{ref}};$$

(9) $$\text{RMSE}=\sqrt{\frac{1}{N}\sum_{i=1}^{N}{\text{residuals}}^{2}}.$$

Here, value_est_ is the mean estimated value per voxel in a region of interest (ROI) for each insert, value_ref_ the reference value obtained either from manufacturer information (RED and EAN) or water column measurements (RSP); *N* is the number of inserts of each phantom.

## 3 Results

From the analyzed 105 energy pairs, we report here the results for the energy pair of 60 keV and 180 keV, which showed the overall best accuracy of EAN and RSP estimation for the calibration Gammex phantom inserts. RED was less sensitive to the choice of energy pair.

### 3.1 RED, EAN, and RSP accuracy

**Figure 1:**
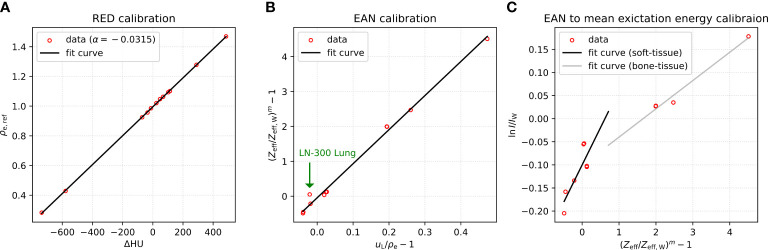
**(A)** RED calibration, **(B)** EAN calibration, and **(C)** EAN to mean excitation energy calibration based on measured CT numbers of Gammex phantom at virtual monoenergies 60 and 180 keV.

**Figure 1** shows calibration curves from fitting procedures corresponding to equations 1 (**Figure 1A**), 2 (**Figure 1B**), and 4 (**Figure 1C**) using measured CT numbers of Gammex inserts from VMIs at 60 and 180 keV. RED was well fitted with a coefficient of determination *R* ^2 =^ 0.9999. For fitting EAN as plotted in **Figure 1B**, *R* ^2^ was 0.9934. The outlier is the *LN-300 Lung* insert with the largest residuals. The calibration for the conversion of EAN to mean excitation energy yielded *R* ^2 =^ 0.6974 for soft-tissue inserts and *R* ^2 =^ 0.9804 for bone-tissue inserts.

**Figure 2:**
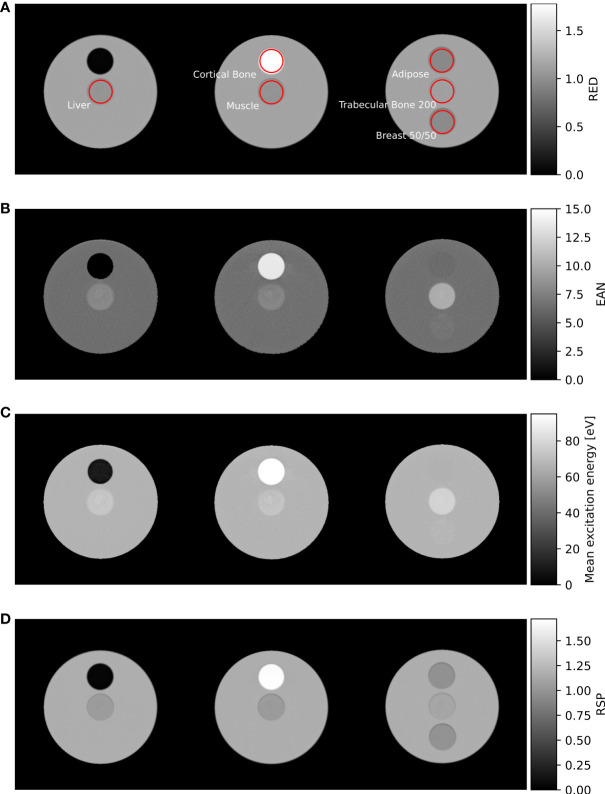
**(A)** RED, **(B)** EAN, **(C)** mean excitation energy, and **(D)** RSP maps of the CIRS (evaluation) phantom, acquired from VMIs at 60 and 180 keV. Insert ROIs used for data analysis are shown in red.

The fit parameters obtained are listed in **Table 2**. Using the acquired fit parameters, the measured CT numbers were converted into RED (**Figure 2B**), EAN (**Figure 2A**), and mean excitation energy (**Figure 2C**) for both the calibration and evaluation phantoms. **Figure 2** presents the maps of these quantities, along with RSP (**Figure 2D**) calculated from RED and mean excitation energy, at three different slices of the CIRS (evaluation) phantom.

**Table 2:** Fit parameters obtained using VMIs of Gammex (calibration) phantom at 60 and 180 keV.

	Soft-tissue		Bone-tissue
*a*		0.9737
*b*		0.9955
*α*		-0.0315
*γ*_*L*_		9.7653
*γ*_0_		-0.0448
*C*_1_	0.1620		0.0612
*C*_0_	0.0999		0.1011

**Figure 3:**
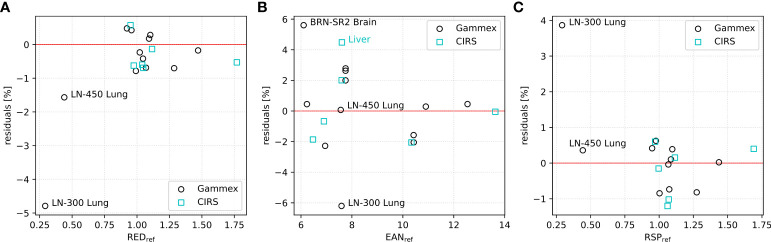
Residuals of mean calculated **(A)** RED, **(B)** EAN, and **(C)** RSP values within insert ROIs for Gammex (calibration, in black) and CIRS (evaluation, in cyan) phantoms using 60 and 180 keV VMIs.

The residuals of RED, EAN, and RSP evaluated for both calibration and evaluation inserts within the ROIs shown in red in **Figure 2A** are presented in **Figure 3**. As shown in the left-most plot (**Figure 3A**), the estimation of RED demonstrated an accuracy within 0.8% except -1.6% and -4.8% for the two lung inserts. For EAN, an agreement with reference values within 2.8% can be observed for most of the inserts, whereas for *Liver* (CIRS), LN-300 Lung (Gammex), and *BRN-SR2 Brain* (Gammex) the errors exceeded 4.4% (see **Figure 3B**). For RSP, the majority of the inserts had residuals below 1.0%; two inserts, *Liver* and *Muscle* (CIRS), exhibited values slightly above 1.0%. The *LN-300 Lung* insert had the highest error (-3.9%, see **Figure 3C**).

The corresponding RMSEs averaged over phantom inserts are given in **Table 3**. As the RSP value of the Gammex *Muscle* insert was not measured (25), the RMSE calculation of RSP for the Gammex (calibration) phantom excluded the *Muscle* insert. The two lung-mimicking inserts (*LN-450 Lung* and *LN-300 Lung*) showed relatively worse results as also observed in previous studies (18, 25, 26). Therefore, the RMSEs for the Gammex phantom were also calculated excluding lung inserts. Additionally, RMSEs of RSP acquired at a state-of-the-art dual-source DECT scanner (SOMATOM Definition FORCE, *Siemens Healthineers, Forchheim, Germany*) at 150 kVp with Sn filtration and 90 kVp in previous studies (11, 25) are presented in **Table 3**.

**Table 3:** RMSEs of RED, EAN, and RSP for the calibration (Gammex) and evaluation (CIRS) phantoms using VMIs at 60 and 180 keV.

		PCCT		DECT	
				Hudobivnik et al. (25)	Dedes et al. (11)
CTDI_vol_ [mGy]		40		20	35.7
Phantom	RED	EAN	RSP	RSP	RSP
Gammex	1.52%	2.90%	1.27%	1.1%	n. a.
(excluding lung inserts)	0.49%	2.50%	0.54%	n. a.	n. a.
CIRS	0.56%	2.32%	0.71%	0.8%	0.68%

RMSEs calculated excluding two lung inserts, LN-450 Lung and LN-300 Lung, for the Gammex phantom are also given. The right-most two columns show RMSEs of RSP obtained with DECT along with the respective imaging dose in previous works (11, 25).

Detailed information about measured CT numbers and reference and calculated RED, EAN, and RSP values of the studied inserts are listed in **Table 4**.

**Table 4:** Mean and standard deviation of CT numbers for Gammex and CIRS inserts from 60 keV (HU_L_) and 180 keV (HU_H_) VMIs, along with the mean and standard deviation of the calculated RED (*ρ*_e,cal_), EAN (*Z*_eff,cal_), and RSP values for all inserts.

Insert	HU_L_	HU_H_	*ρ*_e,ref_	*ρ*_e,cal_	*Z*_eff,ref_	*Z*_eff,cal_	RSP_ref_	RSP_cal_
**Gammex**								
BR-12 Breast	-61 ± 5	-34 ± 5	0.957	0.961 ± 0.004	6.95	6.8 ± 0.2	0.973	0.979 ± 0.006
AP6 Adipose	-111 ± 4	-69 ± 4	0.925	0.927 ± 0.004	6.23	6.3 ± 0.2	0.943	0.947 ± 0.005
LN-450 Lung	-563 ± 29	-579 ± 27	0.429	0.433 ± 0.026	7.56	7.6 ± 0.5	0.436	0.438 ± 0.027
LN-300 Lung	-723 ± 14	-737 ± 13	0.282	0.278 ± 0.012	7.59	7.1 ± 1.0	0.272	0.283 ± 0.013
LV1 Liver	89 ± 6	68 ± 5	1.063	1.062 ± 0.005	7.75	7.9 ± 0.2	1.079	1.071 ± 0.006
BRN-SR2 Brain	6 ± 5	49 ± 4	1.047	1.042 ± 0.004	6.10	6.4 ± 0.2	1.064	1.064 ± 0.005
Muscle	48 ± 5	23 ± 5	1.020	1.019 ± 0.005	7.75	8.0 ± 0.2	n. a.	1.027 ± 0.006
CT Solid Water	11 ± 5	-13 ± 5	0.986	0.984 ± 0.005	7.75	8.0 ± 0.2	1.000	0.992 ± 0.006
CB2-50% CaCO3	1165 ± 10	463 ± 9	1.469	1.468 ± 0.008	12.54	12.6 ± 0.1	1.434	1.434 ± 0.010
CB2-30% CaCO3	611 ± 7	279 ± 8	1.278	1.277 ± 0.008	10.90	10.9 ± 0.1	1.279	1.269 ± 0.009
B200 Bone Mineral	314 ± 6	107 ± 7	1.101	1.106 ± 0.006	10.43	10.2 ± 0.1	1.100	1.104 ± 0.007
IB Inner Bone	306 ± 6	95 ± 6	1.093	1.095 ± 0.006	10.42	10.3 ± 0.1	1.092	1.093 ± 0.007
**CIRS**								
Liver	69 ± 5	46 ± 4	1.050	1.041 ± 0.004	7.61	7.9 ± 0.1	1.060	1.049 ± 0.005
Cortical Bone	1893 ± 8	749 ± 8	1.769	1.760 ± 0.008	13.63	13.62 ± 0.05	1.690	1.697 ± 0.009
Muscle	52 ± 6	39 ± 6	1.042	1.034 ± 0.005	7.60	7.8 ± 0.2	1.057	1.044 ± 0.007
Adipose	-81 ± 3	-40 ± 3	0.956	0.955 ± 0.003	6.47	6.3 ± 0.2	0.970	0.976 ± 0.003
Trabecular Bone 200	316 ± 6	116 ± 6	1.116	1.114 ± 0.006	10.34	10.1 ± 0.1	1.112	1.114 ± 0.006
Breast 50/50	-51 ± 3	-26 ± 3	0.976	0.969 ± 0.003	6.90	6.9 ± 0.1	0.988	0.986 ± 0.004

Reference values are also given.

### 3.2 Comparison between different low- and high-energy pairs

**Figure 4:**
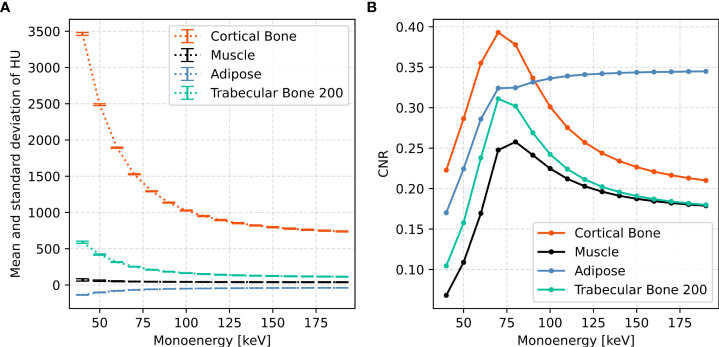
**(A)** Mean (HU_mean_ ) and standard deviation (HU_std_ ) of CT numbers and **(B)** the corresponding CNR at monoenergies ranging from 40 to 190 keV in 10 keV increments for four CIRS inserts: *Liver*, \textit{Cortical Bone}, *Muscle*, and *Adipose*. CNR was calculated by CNR=(HU_mean_/1000+1)/HU_std_.

**Figure 4A** shows mean and standard deviation of CT numbers at all the monoenergies for four exemplary evaluation (CIRS) inserts with the highest and the lowest density: *Cortical Bone*, *Muscle*, *Adipose*, and *Trabecular Bone 200*. The corresponding contrast-to-noise ratios (CNRs) are plotted in **Figure 4B**.

Regardless of the chosen low- and high-energy pair, the RMSE of RED mostly (102 out of 105 pairs) ranges from 0.55% to 0.56% for the CIRS (evaluation) phantom and from 1.51% to 1.54% for the Gammex (calibration) phantom. The few exceptions with a higher RMSE are listed in **Table 5**.

**Table 5:** RMSEs of RED for different monoenergy pairs.

Phantom	Low energy	High energy	RMSE of RED
	[keV]	[keV]	
Gammex	**60**	**180**	**1.52%**
	160	180	1.65%
	160	190	1.65%
	170	190	1.91%
	Other cases		1.51% - 1.54%
CIRS	**60**	**180**	**0.56%**
	160	180	0.58%
	160	190	0.58%
	170	190	0.71%
	Other cases		0.55% - 0.56%

The low- and high-energy pair 60 and 180 keV (in bold) was the pair with which the overall best EAN and RSP accuracy was obtained for the calibration (Gammex) phantom. The other three energy pairs listed in this table had the highest errors. The rest showed similar degree of RED accuracy.

**Figure 5:**
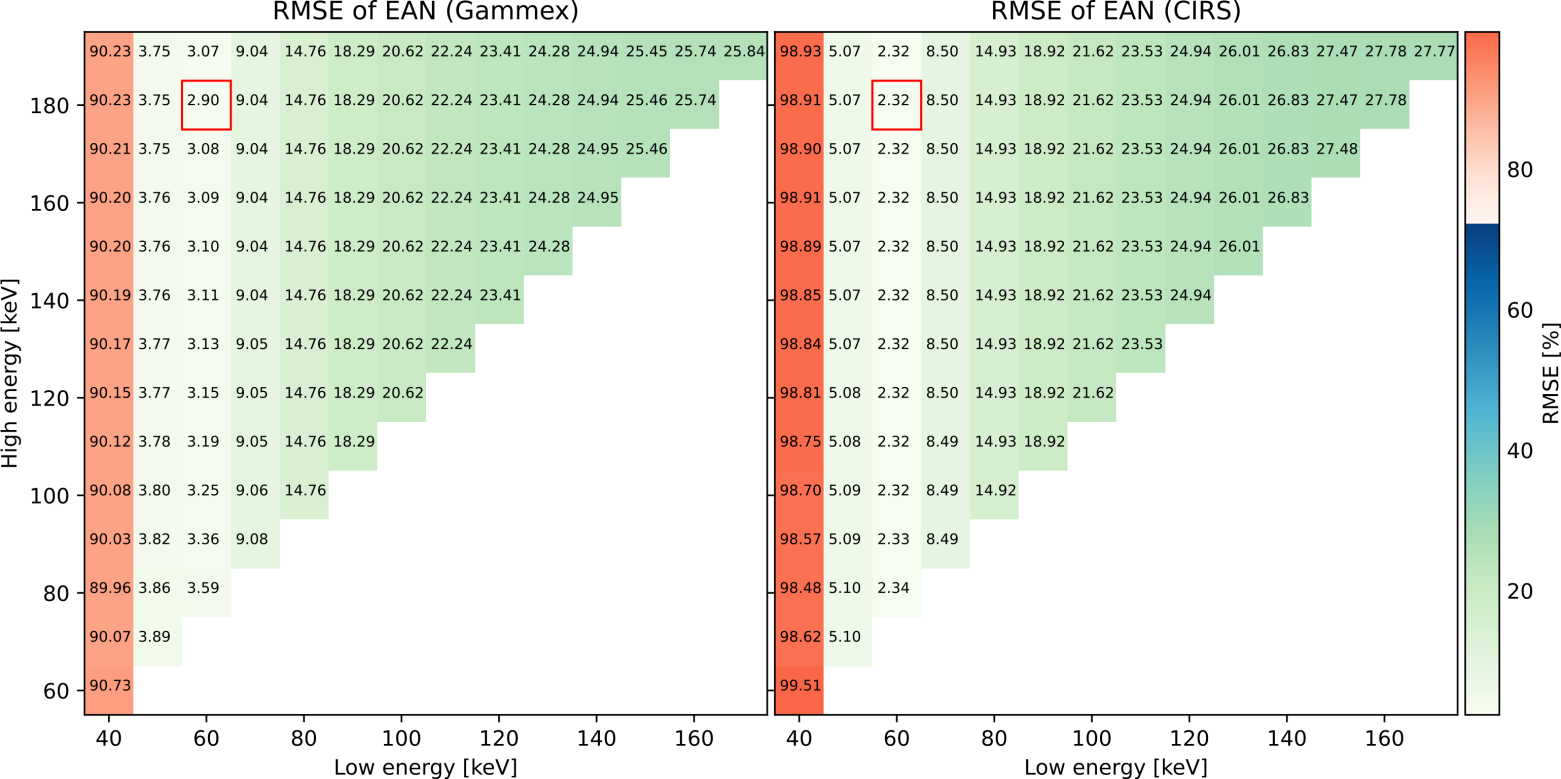
RMSEs of EAN for all the monoenergy pairs.

**Figure 5** presents RMSEs of EAN evaluated for both phantoms using different low- and high-energy pairs of VMIs. RMSE values are displayed on a background of different shades of colors corresponding to the value. Similar patterns can be observed for the two phantoms: energy pairs sharing the same low-energy VMIs had nearly the same RMSE, although a minimum could still be identified. Errors of EAN using 40 keV low-energy VMIs were remarkably high, around 90% and 99% for the Gammex and the CIRS phantom, respectively. The low energy 60 keV had the best EAN accuracy, 50 keV the second. The EAN accuracy decreased with an increasing low energy. The lowest error, 2.90% for the Gammex phantom and 2.32% for the CIRS phantom, obtained with 60 and 180 keV VMIs, is marked by a red box in both plots.

**Figure 6:**
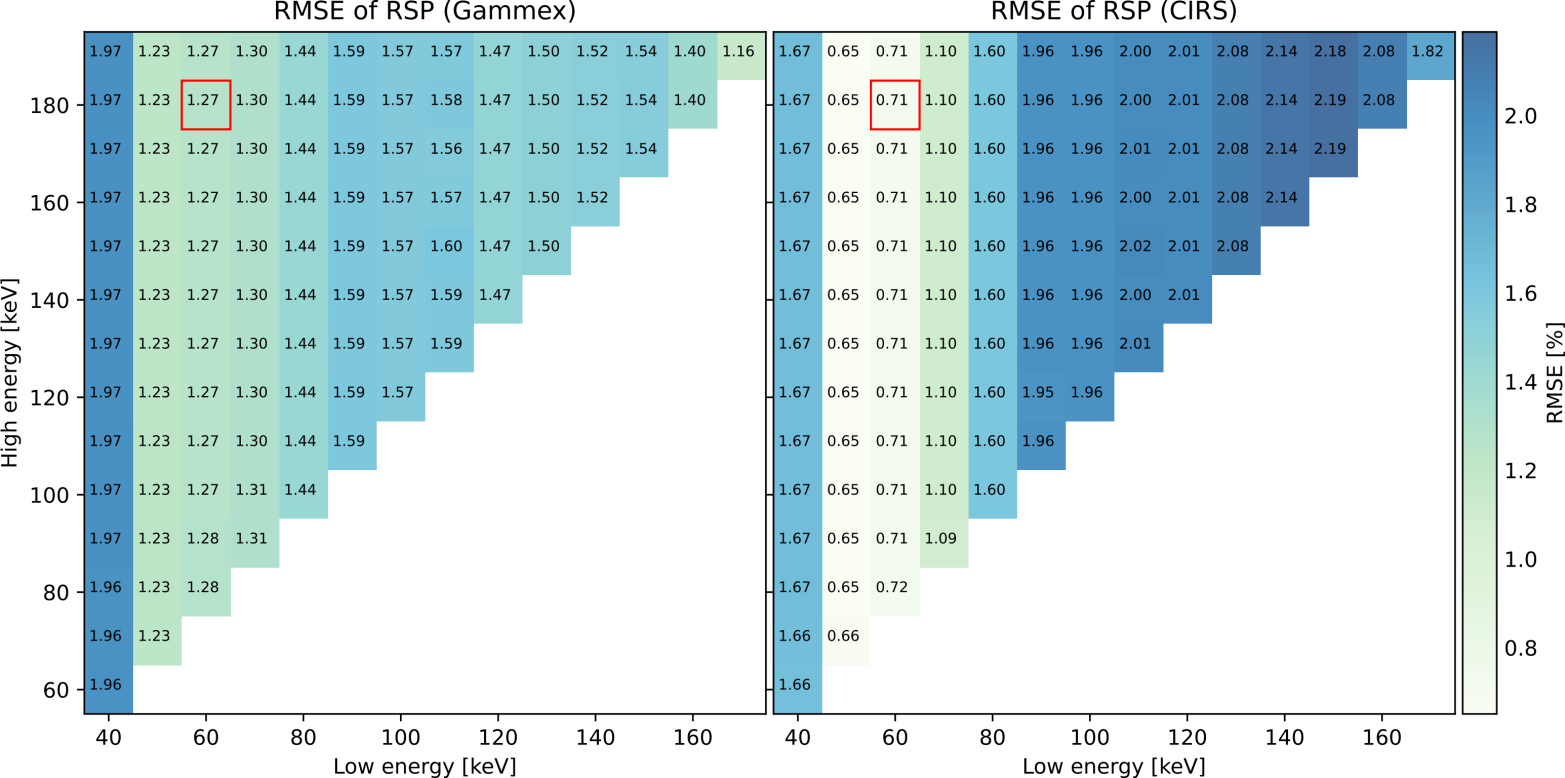
RMSEs of RSP for all the monoenergy pairs.

Like EAN, RMSEs of RSP are shown in **Figure 6**, and similar trends were observed: For the same low energy, RMSE barely varied despite the different choice of high energy. The RSP accuracy using the optimal energy pair 60 and 180 keV, 1.27% for the Gammex phantom and 0.71% for the CIRS phantom, is marked by a red box in both plots.

## 4 Discussion

The low *R* ^2^ for soft-tissue inserts was potentially caused by suboptimal tissue equivalence in terms of the relation between *Z*_eff_ and *ln* *I* for the calibration inserts. It was demonstrated in Yang et al. (27) that the fit showed a higher *R* ^2^ when performed based on tabulated human tissues [see **Figure 3** in Yang et al. (27)].

The Gammex phantom showed higher errors (1.52%) than the CIRS phantom (0.56%) in RED estimation due to the relatively high residuals of lung inserts. The RED RMSE for the calibration (Gammex) inserts was reduced to 0.49% when the lung inserts were excluded.

The EAN estimation accuracy was much worse when using 40 keV as the low-energy VMI than when a higher energy was used. This could arise from the dominant photoelectric absorption at lower energies. As a result, EAN calibration curves were less well fitted, leading to higher errors at 40 keV.

In Hudobivnik et al. (25), Gammex and CIRS inserts were scanned at a dual-source DECT scanner, yielding an RSP RMSE of 1.1% for the Gammex phantom and 0.8% for the CIRS phantom. In (11), the RMSE of RSP was 0.68% considering the same CIRS inserts as studied in this work (cf. **Table 3**). Using PCCT data in this study, comparable accuracy was demonstrated: 1.27% for the Gammex phantom and 0.71% for the CIRS phantom.

Using equation 6, Doolan et al. (28) have shown that the theoretical calculation of RSP using ICRU *I*-values and manufacturer-provided electron densities and elemental compositions leads to errors of 0.96% compared to measurements of Gammex tissue substitutes [see Table 6 in Doolan et al. (28)]. Uncertainties in the insert composition could affect the accuracy of RSP calculation in Doolan et al. (28) as well.

The optimal pair of VMIs, 60 keV low energy and 180 keV high energy, was determined solely on the basis of the calibration (Gammex) phantom. The use of VMIs to assess RED, EAN, and RSP allows analyzing data without access to the underlying vendor processing software, whereas access to basis images, which were weighted by the scanner software to yield VMIs, would be an alternative to using VMIs. The same formalism as used in this study would be applicable to a pair of basis images.

In this work, two sets of tissue-equivalent materials were used for calibration and evaluation. The same calibration method was used for DECT conversion in Niepel et al. (8), where RSP estimation results in animal tissues have been shown more accurate than in tissue-equivalent materials.

A high dose (CTDI_vol_=40 mGy ), comparable to previous DECT studies (11), was used in the scans with the intention of observing the performance limits. It would be worth performing tests at lower doses in future studies. The analysis was limited to data acquired at a tube voltage of 120 kVp, a more extensive study with comparison to measurements at 140 kVp tube voltage would be of interest, as well as studying the impact of the number of energy bins and basis images.

The results reported in this work were obtained in the first year of operation of the scanner. Forthcoming studies will address reproducibility assessment and possible improvements due to latest upgrades by the manufacturer, and compare performance to DECT not only in terms of RSP accuracy but also in terms of noise and resolution performance. Additionally, the accuracy of tissue composition estimation compared to DECT will be investigated (29).

## 5 Conclusion

We performed measurements of tissue-mimicking inserts at a first-generation clinical PCCT scanner and evaluated the accuracy of RSP estimation from VMIs provided by the scanner. The optimal pair of VMIs at 60 and 180 keV energies showed comparable accuracy to that obtained at DECT scanners. The RMSEs of RSP for the calibration and the evaluation phantom were 1.27% (0.54% when lung inserts were excluded) and 0.71%, respectively.

## Data availability statement

The mean HU values of the optimal VMI pair necessary to reproduce our results can already be found in **Table 4**. Requests to access the datasets should be directed to GH, Guyue.Hu@physik.uni-muenchen.de; KP, Katia.Parodi@physik.uni-muenchen.de.

## Author contributions

GL, KP, FS, and TK contributed to design of the study. CK, GL, and FS were involved in the data acquisition. FR supported the data reconstruction. KN supported the data analysis. GH analyzed the data. GH, KN, KP, GL, CK, and MW critically assessed the results. GH, GL, and KP contributed to the writing. All authors provided feedback to the manuscript. All authors contributed to the article and approved the submitted version.

## Funding

This work was funded by the German Research Foundation (DFG) within the Research Training Group GRK 2274 (grant number 299102935).

## Acknowledgments

The authors acknowledge Dr. Bernhard Schmidt from Siemens Healthineers for answering questions related to the scanner.

## Conflict of interest

FS and University Hospital Augsburg have received speaker honoraria from Siemens Healthineers.

The remaining authors declare that the research was conducted in the absence of any commercial or financial relationships that could be construed as a potential conflict of interest.

## Publisher’s note

All claims expressed in this article are solely those of the authors and do not necessarily represent those of their affiliated organizations, or those of the publisher, the editors and the reviewers. Any product that may be evaluated in this article, or claim that may be made by its manufacturer, is not guaranteed or endorsed by the publisher.
